# PU.1 dictates β-amyloid-induced TREM2 expression upregulation in microglia in a transgenic model of Alzheimer’s disease

**DOI:** 10.3389/fnagi.2025.1537388

**Published:** 2025-05-01

**Authors:** Zhen Wei, Xiaodong Pan, Xiaoli Cui, Jing Zhang, Xiaoman Dai, Yuqi Zeng, Xiaochun Chen

**Affiliations:** ^1^Department of Neurology, Fujian Provincial Hospital, Shengli Clinical Medical College of Fujian Medical University, Fuzhou University Affiliated Provincial Hospital, Fuzhou, China; ^2^Department of Neurology, Fujian Medical University Union Hospital, Fuzhou, China; ^3^Fujian Institute of Geriatrics, Fujian Medical University Union Hospital, Fuzhou, China; ^4^Institute of Clinical Neurology, Fujian Medical University, Fuzhou, China; ^5^Key Laboratory of Molecular Neurology, Institute of Neuroscience, Fujian Medical University, Fuzhou, China; ^6^Key Laboratory of Brain Aging and Neurodegenerative Diseases, Fujian Medical University, Fuzhou, China; ^7^Department of Geriatric, The People’s Hospital Affiliated to Fujian University of Traditional Chinese Medicine, Fuzhou, China

**Keywords:** TREM2, PU.1/Spi1, β-amyloid, microglia, Alzheimer’s disease, 5 × FAD

## Abstract

**Introduction:**

Microglial dysfunction is characteristic of Alzheimer’s disease (AD), with triggering receptor expressed on myeloid cells 2 (TREM2) and transcription factor PU.1 playing crucial roles. However, the relationship between TREM2 and PU.1 remains unclear.

**Methods:**

We investigated TREM2 and PU.1 expression patterns in the 5×FAD mouse AD model. Experimental approaches included quantitative PCR, western blotting, immunofluorescence staining, chromatin immunoprecipitation, and luciferase reporter assays to examine the interaction between PU.1 and TREM2. The phagocytic function of microglial cells was evaluated using Aβ42 and Nile red fluorescent microsphere phagocytosis assays.

**Results:**

TREM2 and PU.1 expression significantly correlated with brain β-amyloid (β) deposition. PU.1 directly interacted with the TREM2 promoter region, promoting its transcription and potently impacting microglial phagocytosis. PU.1 overexpression amplified TREM2 expression, while PU.1 knockdown reduced it.

**Discussion:**

Our findings reveal a novel regulatory mechanism where PU.1 directly modulates TREM2 transcription in activated microglia during AD progression. These insights highlight the potential of TREM2 and PU.1 as therapeutic targets in AD treatment.

## 1 Introduction

As the most prevalent form of dementia among individuals over the age of 60, Alzheimer’s disease (AD) is characterized by a chronic and progressive decline in cognitive function (Jucker and Walker, 2023; Liu et al., 2019; Monteiro et al., 2023; Scheltens et al., 2021; Twarowski and Herbet, 2023; Yan et al., 2022). The disease features distinct pathologies, including abundant extracellular β-amyloid (Aβ) plaques, intracellular neurofibrillary tangles comprising hyperphosphorylated tau protein, extensive neuron loss, and synaptic dysfunction (Graff-Radford et al., 2021; Rostagno, 2022; Serrano-Pozo et al., 2021; Yeung et al., 2023). Recent advancements in high-throughput sequencing technologies have facilitated the identification of numerous AD susceptibility loci. These genes include *CLU, CR1*, *CD33*, *EPHA1*, and *TREM2*, which are involved in immunity, and *APOE* and *ABCA7*, which are involved in lipid processing (Kim et al., 2023; Raulin et al., 2022; Zhao et al., 2015). Notably, the R47H missense mutation in triggering receptor expressed on myeloid cells 2 (*TREM2*) has been shown to significantly increase the risk of AD, with an odds ratio comparable to that of the APOE ε4 allele (Qin et al., 2021; Silvin et al., 2022; Ulland et al., 2017; Zhao et al., 2022c).

Triggering receptor expressed on myeloid cells 2, a member of the immunoglobulin (Ig) superfamily, is expressed primarily by microglia in the central nervous system (Gratuze et al., 2023; Jain et al., 2023; Nugent et al., 2020; Wang et al., 2022; Zhong et al., 2023). Extensive research has revealed that mutations in or functional loss of TREM2 may contribute to the onset of AD. Overexpression of TREM2 can attenuate neuropathology and reverse spatial cognitive deficits in APPswe/PS1dE9 mice, a model of AD (Jiang et al., 2014). Conversely, the loss of TREM2 function can exacerbate tau pathology and neurodegenerative changes in P301S tau transgenic mice (Jiang et al., 2015). Further, the AD-associated R47H variant has been found to impair microglial recognition of anionic lipids, such as phosphatidylserine, sulfatides, and sphingomyelin-lipids, exposed to neuronal damage. This impaired recognition leads to microglial dysfunction and compromised clearance of Aβ plaques (Wang et al., 2015).

The full-length human TREM2 consists of an extracellular domain, a transmembrane domain, and a short cytoplasmic tail, lacking signaling motifs (Bouchon et al., 2000). TREM2 signal transduction is mediated by the DNAX-activating protein of 12 kDa (DAP12), which contains a single immunoreceptor tyrosine-based activation motif (ITAM). The TREM2–DAP12 complex orchestrates a spectrum of biological functions, including phagocytosis, actin reorganization, chemotaxis, and immunoregulation (Fracassi et al., 2023; Li et al., 2024; Xue et al., 2022; Yerlikaya et al., 2022). However, the regulatory mechanisms that govern TREM2 remain unclear.

Interestingly, the E26 transformation-specific (ETS) family transcription factor PU.1/Spi1 is also highly expressed in microglia but not in astrocytes or neurons (Smith et al., 2013a). Previous studies have suggested that PU.1 and C/EBPα modulate microglial activation and proliferation during neurodegenerative processes and appear to be upregulated in human AD and prion diseases (Greter and Merad, 2013). In the presence of PU.1 siRNA, microglia exhibit reduced phagocytosis of Aβ1-42 peptides, accompanied by a significant downregulation of cell viability-related genes (Smith et al., 2013b). Given the functional parallels between PU.1 and TREM2, there is a potential association, and it is worthwhile to explore the regulatory mechanisms underlying TREM2 expression upregulation in chronic neurodegenerative diseases.

In the present study, we demonstrated that PU.1 plays a decisive role in Aβ-induced TREM2 upregulation by binding directly to the enhancer and promoter regions of the *TREM2* gene. Our findings reveal a novel mechanism by which the transcription factor PU.1 acts as a critical regulator in switching the reactive phenotypes of microglia during AD progression.

## 2 Materials and methods

### 2.1 Animals

Male 5 × FAD APP/PS1 double transgenic B6/eSJL mice were procured from Jackson Laboratory (stock number 034848-JAX, Bar Harbor, ME, United States). These 5 × FAD mice harbor mutations at APP K670N/M671L, I716V, V717I, and PS1 M146L, L286V, which, under the regulation of the neuron-specific murine Thy-1 promoter, can produce an abundance of Aβ42 peptides (Oakley et al., 2006). The mice were maintained in an environment with a 12- h light/dark cycle and allowed free access to water and food. All of the experimental procedures involving mice were approved and conducted in accordance with the guidelines of the Institutional Animal Care and Use Committee at Fujian Medical University (IACUC FJMU 2021-0272) and per international standards for the ethical treatment of animals.

### 2.2 Primary microglia and BV2 cell culture

Primary microglia were isolated from the brains of the mice at postnatal day 1. In brief, the cortex and hippocampus were excised from newborn littermates and dissociated in papain solution. The samples were centrifuged, resuspended in DMEM/F12 supplemented with 10% fetal bovine serum (FBS), and seeded in 75 cm^2^ culture flasks. The culture medium was changed every 3 days. After 14 days, the microglia were dislodged from the mixed glial cultures via an orbital shaker set at 200 rpm for 60 min at 37°C. The BV-2 immortalized mouse microglial cell line was cultured in DMEM/high-glucose medium enriched with 5% FBS, 100 units/ml penicillin, and 100 μg/ml streptomycin and incubated at 37°C in a humidified atmosphere containing 5% CO2 and 95% air. BV2 cells were passaged upon reaching 70%–80% confluency.

### 2.3 PU.1 silencing of RNA and PU.1 overexpression

siRNAs targeting the murine Spi1 gene were used to inhibit the endogenous expression of PU.1. The effective siRNA sequence (AATGCATGACTACTACTCCTT) was used to construct a short hairpin RNA (shRNA) as previously described (Zou et al., 2007). This shRNA sequence, along with its control counterpart (TTCTCCGAACGTGTCACGT), was cloned and inserted into the GV248 vector (hU6-MCS-Ubiquitin-EGFP-IRES-puromycin). The lentiviral vector was assembled, concentrated, and titrated by a commercial service provider (Shanghai Genechem, China), with viral particles designated as LV-PU.1-RNAi with a titer of 4E + 8 IU/ml. Lentiviral transduction was performed following the manufacturer’s recommended protocol. Briefly, BV2 microglia were seeded at 25%–30% confluence in 6-well plates and allowed to attach for 12 h. The cells were then assigned to one of three groups: the Lv-shRNA (transduced with PU.1-targeting shRNA virus) group, the Lv-CTL (transduced with control shRNA virus) group, and the blank (untreated) group. Transduction was performed at a multiplicity of infection (MOI) of 100 in the presence of 8 μg/ml polybrene (Shanghai Genechem, China). After a 16 h incubation at 37°C, the supernatant was removed, and the cells were further cultured in complete growth medium. On the third day after transduction, the cells were passaged and exposed to selection medium containing 4.5 μg/ml puromycin for 48 h. The knockdown efficiency was evaluated via flow cytometry (BD Accuri C6, United States) and fluorescence microscopy (LSM780, Carl Zeiss), confirming the approximate 95% efficacy in the gene-silenced phenotype.

For the overexpression of PU.1, the coding sequence for mouse Sfpi1 (NM_011355) was amplified, cloned, and inserted into the GV358 vector (Ubi-MCS-3FLAG-SV40-EGFP-IRES-puromycin). The corresponding negative control was an empty GV358 vector. The lentiviral vector system construction, cell transduction procedures, and establishment of the PU.1 overexpression model were conducted as described for PU.1 RNA silencing.

### 2.4 Quantitative real-time PCR

Fresh hippocampal tissues were procured and subjected to total RNA isolation using TriPure Isolation Reagent (Roche, Germany) per the manufacturer’s protocol. The RNA extraction process for primary cultured microglia and the microglial BV-2 cell line was conducted as described above. After extraction, first-strand cDNA was synthesized from 1 μg of the isolated total RNA using a Transcriptor First Strand cDNA Synthesis Kit (Roche, Germany). Quantitative real-time PCR (qRT–PCR) was executed on a StepOne™ Real-Time PCR System (Applied Biosystems, Foster City, CA) utilizing SYBR Green Master Mix (Roche) for the fluorescent quantification of the amplified products. The thermal cycling conditions for qRT–PCR were as follows: an initial denaturation step at 95°C for 10 min, followed by 40 cycles of denaturation at 95°C for 15 s, and annealing/extension at 60°C for 1 min. The change in gene expression was calculated via the ΔΔCt method, with *gapdh* serving as the reference gene for normalization. The sequences of the primer pairs used are as follows: *pu.1* (NM_011355.1; FW, 5′-CAGAAGGGCAACCGCAAGAA-3′; RV, 5′-GCCGCTGAACTGGTAGGTG-3′); *trem2* (NM_031254; FW, 5′-GCCTTCCTGAAGAAGCGGAA-3′; RV, 5′-GAGTG ATGGTGACGGTTCCA-3′), *gapdh* (NM_008084.2; FW, 5′- CAGTGGCAAAGTGGAGATTGTTG-3′; RV, 5′-CTCGCTC CTGGAAGATGGTGAT-3′).

### 2.5 Western blotting

Proteins were extracted from the hippocampal tissues of 5 × FAD and wild-type mice to evaluate the expression of TREM2 and PU.1. After treatment, the BV2 cells were washed with ice-cold PBS and lysed for 25 min in lysis buffer. The lysates were then centrifuged, and the supernatants were collected. Proteins (60 μg from brain tissues and 30 μg from cells) were denatured at 100°C for 5 min in SDS sample loading buffer. Proteins were then resolved via 8% or 10% SDS–PAGE and electroblotted onto polyvinylidene difluoride (PVDF) membranes at 200 mA for 120 min via a Bio-Rad transfer system (Bio-Rad Laboratories, Hercules, CA). The membranes were then blocked for 2 h at room temperature using Odyssey blocking buffer and incubated with the following primary antibodies: rabbit polyclonal anti-TREM2 (1:250, Santa Cruz Biotechnology, CA, United States), PU.1 (1:200, Santa Cruz Biotechnology, CA), β-actin (1:2,000, Abcam), and α-Tubulin (1:10,000, Abcam). This incubation was performed in 2.5% BSA diluted in TBST overnight at 4°C.

### 2.6 Immunohistochemistry

Immunohistochemistry (IHC) was performed following previously established protocols (Pan et al., 2011). In brief, sections (40 μm thick) were treated with 3% hydrogen peroxide for 10 min to quench endogenous peroxidase activity. The sections were subsequently rinsed in Tris-buffered saline (TBS) and then incubated in a blocking solution comprising TBS with 0.3% Triton X-100, 0.25% bovine serum albumin (BSA), and 5% normal goat serum (NGS) at room temperature for 1.5 h. The sections were incubated in TBS containing 0.25% BSA, 2% NGS, 0.3% Triton X-100, and primary anti-PU.1 antibody (1:400, Santa Cruz Biotechnology, United States) for antigen detection at 4°C under gentle agitation for 48 h. The samples were then incubated with biotinylated secondary antibodies (1:600, Vector Laboratories, Burlingame, CA, United States) and the Vector Elite ABC-peroxidase complex. Immunoreactivity was visualized using diaminobenzidine (DAB) as the chromogen. Images were acquired under an Olympus BX-51 microscope (Olympus, Japan).

### 2.7 Immunofluorescence analysis

Double immunofluorescence of free-floating tissue sections was initiated with thorough washing in TBS. The sections were then subjected to a blocking solution comprising TBS supplemented with 0.3% Triton X-100, 0.25% bovine serum albumin (BSA), and 5% normal donkey serum (NDS) at room temperature for 2 h. The slices were then incubated with either Iba1 (1:1000; Wako Pure Chemical Industries) and 6E10 (1:5000; Covance), Iba1 and TREM2 (1:100, R&D Systems), or TREM2 and PU.1 (1:300, Cell Signaling) antibodies followed by species-specific Alexa Fluor secondary antibodies (1:1000; Life Technologies). The sections were then mounted with ProLong Gold antifade reagent (Invitrogen). Confocal images were captured under an LSM 780 META microscope (Carl Zeiss). A total of 12–20 sequential slices, each separated by 1 μm, were imaged and compiled into z-stacks for analysis.

BV2 cells and primary microglia were fixed with 4% freshly prepared paraformaldehyde for 10 min, followed by three rinses with phosphate-buffered saline (PBS). BV2 cells and primary microglia were subsequently blocked for 1 h in a solution of 5% NDS, 0.2% Triton X-100, and 0.25% BSA in PBS. Subsequently, the sections were incubated overnight at 4°C with the following primary antibodies: TREM2 (sheep anti-TREM2, R&D Systems) and PU.1 (1:300, rabbit anti-PU.1, Cell Signaling). The cells were then treated with an appropriate Cy3-conjugated secondary antibody targeting sheep IgG or an Alexa Fluor 594-conjugated secondary antibody targeting rabbit IgG. After three washes in PBS with Tween 20 (PBST), the nuclei were counterstained with DAPI. Finally, the samples were mounted with ProLong Gold antifade reagent (Invitrogen) and visualized under a confocal laser scanning microscope with a range of objectives (Carl Zeiss).

### 2.8 Phagocytosis assay

BV2 microglia were categorized into four experimental groups: those transduced with TREM2-targeting shRNA (TREM2 Lv-shRNA), those transduced with PU.1-targeting shRNA (PU.1 Lv-shRNA), control lentivirus (Lv-CTL), and a non-transduced blank control. Fibrillar Aβ(1–42) preparation and the subsequent phagocytosis assay were conducted as previously described (Pan et al., 2011). In brief, lyophilized Aβ (1–42) peptide was initially rendered monomeric by dissolving it to a concentration of 1 mM in 100% hexafluoroisopropanol (HFIP). To promote fibril formation, Aβ (1–42) was then diluted in sterile Milli-Q water and incubated at 37°C for 1 week. On the day the assay was performed, the cells were exposed to fluorescent microspheres for 30 min or, alternatively, to Hilyte-555-labeled fibrillar Aβ (1–42) (1.0 μM) for 60 min. Subsequently, the cells were fixed with 4% paraformaldehyde (PFA) and visualized with Alexa488-conjugated phalloidin, which specifically stains F-actin. Five randomly selected fields per coverslip were examined, and the mean fluorescence intensity was quantified with Zen software (Zeiss), with the results normalized to those of the untreated control group. The phagocytic efficiency was assessed as previously described (Koenigsknecht and Landreth, 2004).

### 2.9 Chromatin immunoprecipitation (ChIP) qPCR assay

Microglial BV2 cells were prepared for chromatin immunoprecipitation (ChIP) following the protocol established by Millipore (Temecula, CA, United States). In brief, the cells underwent formaldehyde-mediated cross-linking and were subsequently lysed. Fragmentation of genomic DNA was achieved through sonication. For the immunoprecipitation (IP) of the cross-linked protein-DNA complexes, we used an anti-PU.1 antibody from Santa Cruz Biotechnology and a normal rabbit IgG from Cell Signaling Technology as a control. Quantitative PCR was performed to quantify the ChIP signals using the StepOne™ Real-Time PCR System from Applied Biosystems. We calculated relative enrichment by normalizing the percentage of input DNA to that of the controls. The specific primer pairs for the *TREM2* promoter region and UTR were as follows: UTR: FW, 5′- CCCTCCTCCACCAAGACT –3′; RV, 5′- AAGTGCAGAAGTTGACAGAC –3′); Region 1: FW, 5′- CCATCTAGGCCTTAACAT –3′; RV, 5′- AAAT CACTGGACAGGAAC –3′); Region 2: FW, 5′- AAGAAAA GACTGAGCTGTG –3′; RV, 5′- CTGTAGGCAGAAAGGGAG –3′); Region 3: FW, 5′- AAATGAGGCTCTGCAAGGA –3′; RV, 5′- GCTGTGATTCAAGGAGGGAG –3′).

### 2.10 Luciferase reporter assays

BV2 cells were cultured to 80%–90% confluence after seeding into 6-well plates and subsequently transfected with Lipofectamine 3000 reagent (Invitrogen, Carlsbad, CA, United States) per the manufacturer’s protocol. For the construction of a reporter plasmid harboring the *TREM2* promoter, vector A (a 3,000 bp segment proximal to the transcription start site) or its 3’UTR vector B (a 60 bp DNA fragment representing a portion of the TREM2 3’UTR) were chemically synthesized, subcloned, and inserted into the pGL4.10 vector (Promega, Madison, WI, United States). BV2 cells were seeded into 24-well plates at a density of 1 × 10^5^ cells per well for the transfection assays. Each well was transfected with 1 μg of either vector A or B, both containing the Firefly luciferase reporter gene, with 0.2 μg of the pRL-TK vector (Promega, Madison, WI, United States) encoding Renilla luciferase as an internal control, or with the Firefly luciferase vector alone, as appropriate, via the Lipofectamine 3000 reagent following the manufacturer’s guidelines. At 72 h after transfection, luciferase activities were quantified using the Dual-Luciferase Reporter Assay System (Promega, E1910). The results were normalized and reported as the ratio of Firefly to Renilla luciferase activities to account for variations in transfection efficiency, with the final values presented as relative luciferase activity.

### 2.11 Statistical analysis

The data are presented as the means ± SEMs and were derived from a minimum of three independent experiments. Analysis was performed via GraphPad Prism version 6.01 (GraphPad Software, La Jolla, CA, United States). Data of normality and homoscedasticity were analyzed via one-way or two-way ANOVA (two variables were analyzed in all cases), followed by the Bonferroni *post hoc* correction for multiple comparisons. The partial correlation was analyzed using the Pearson test. *P* < 0.05 was considered to indicate significance.

## 3 Results

### 3.1 TREM2 expression levels are significantly elevated in activated microglia

**FIGURE 1:**
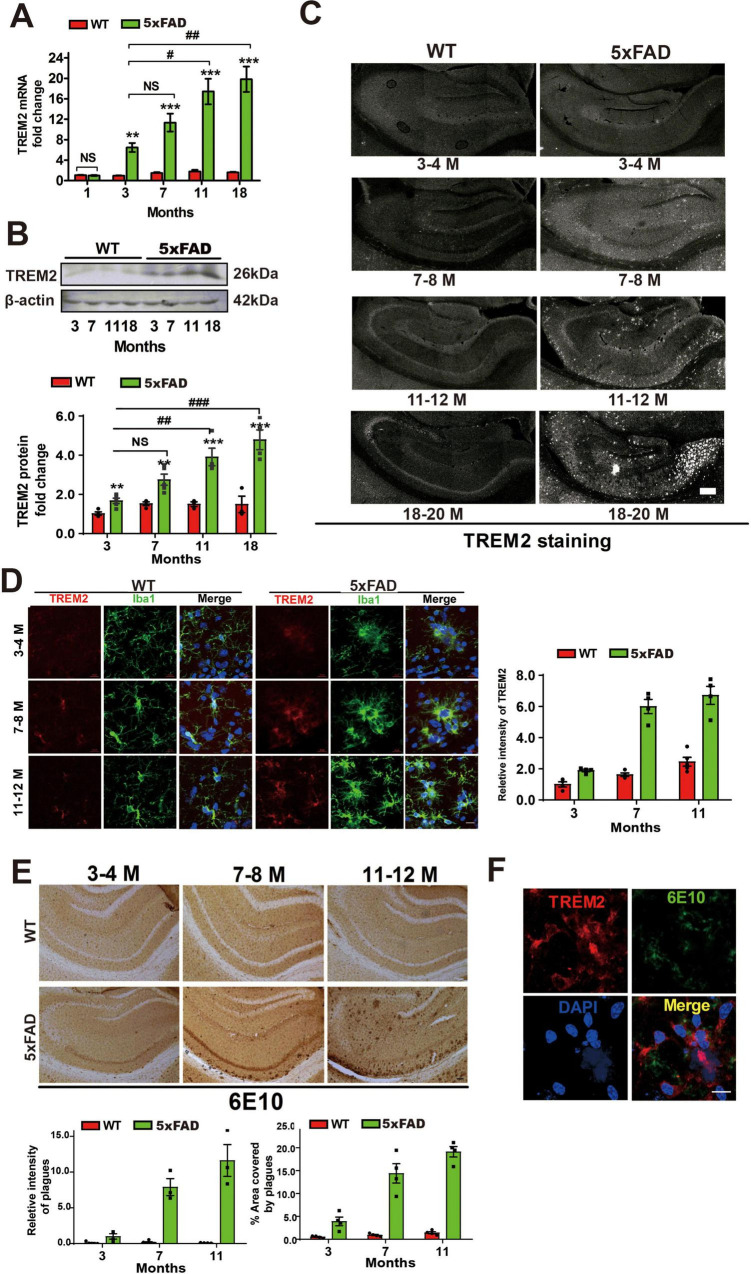
Elevated TREM2 protein and mRNA levels in activated microglia in the hippocampus of 5 × FAD mice during disease progression. **(A)** Analysis of the mRNA expression of TREM2 in the hippocampi of 1 month-old to 18–20 months-old 5 × FAD mice and aged-matched WT mice. The expression of TREM2 mRNA is presented as the mean ± S.E.M. and is shown as the relative expression level according to the 2^Δ^ ^Δ^ ^Ct^ method. The data were analyzed via one-way analysis of variance followed by Bonferroni *post hoc* correction. #*p* < 0.05, ##*p* < 0.01, ###*p* < 0.001, 5 × FAD young group (3–4 months old) vs. other 5 × FAD groups. *p < 0.05, **p < 0.01, ***p × 0.001, WT vs. 5 × FAD. **(B)** Representative western blot of TREM2 in whole hippocampal lysate, expressed as the mean ± S.E.M. **(C)** Representative confocal photomicrographs of the whole hippocampus. Note the increased immunoreactivity of TREM2 (red) in the hippocampus, especially in the CA1 region, of 5 × FAD mice. The expression increased with age. Scale bar = 200 μm. **(D)** Dual-label immunofluorescence image showing TREM2 (red) with microglia (green). Nuclei are stained with DAPI (blue). Notably, the increased immunoreactivity of TREM2 (red) was mostly associated with activated microglia (Iba1, green) rather than with resting microglia. Scale bar = 10 μm. **(E)** Immunohistochemistry (IHC) showing Aβ deposits stained with 6E10 in WT mice and 5 × FAD mice. Scale bar = 100 μm. **(F)** Dual-label immunofluorescence showing that TREM2 (red) is expressed mainly in plaque-associated areas (6E10, green). Scale bar = 10 μm. ns, not significant; TREM2, triggering receptor expressed on myeloid cells-2; WT, wild type; Iba1, ionized calcium-binding adapter molecule 1.

To elucidate the changes in TREM2 expression in 5 × FAD mice, we quantified the mRNA and protein levels of TREM2 in brain tissues. Our analysis revealed that both the mRNA and protein expression levels of TREM2 were significantly greater in 5 × FAD mice than in their age-matched wild-type (WT) counterparts (Figures 1A, B). We observed an age-dependent increase in TREM2 expression: TREM2 levels were markedly elevated in aged 5 × FAD mice (≥ 11 months of age) compared with their younger counterparts (3–4 months of age), whereas at 1 month of age, there was no notable difference in TREM2 mRNA expression levels between 5 × FAD and WT mice (Figure 1A). To gain further insight into the spatial and temporal dynamics of TREM2, we probed its immunoreactivity within the hippocampus. With advancing age, intensified TREM2 staining was noted, which exhibited a distinct spatial and age-dependent pattern, with initial localization to the CA1 region in early life (3–4 months of age) and late spreading throughout the entire hippocampal structure by 18–20 months of age in 5 × FAD mice (Figure 1C).

Subsequent investigations focused on identifying the cellular localization and types of TREM2 expressed. We discovered that the increase in TREM2 expression was predominantly located in activated microglia (Iba-1-positive microglia) (Figure 1D), which also clustered around amyloid plaques (Figure 1F). Available evidence shows that the transgenic AD model displays reactive microgliosis featuring retracted processes and an “amoeboid” morphology, whereas the microglia of WT mice remain in a “resting” state (Oakley et al., 2006). Similarly, we detected gradual and rapid Aβ deposition, primarily in the hippocampal formation, in 5 × FAD mice (Figure 1E). These results demonstrate that TREM2 is selectively augmented in microglia encircling Aβ deposits and exhibits distinctive age-dependent and localized characteristics in 5 × FAD mice.

### 3.2 PU.1 expression is markedly increased in the hippocampus of 5 × FAD mice

**FIGURE 2:**
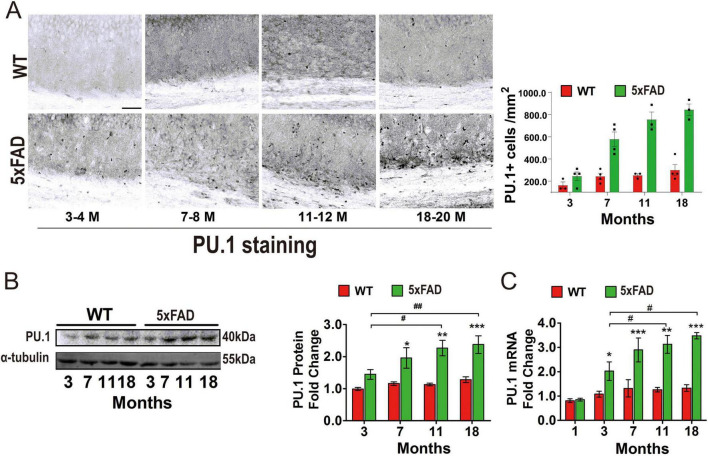
Upregulation of PU.1 protein and mRNA expression in the hippocampus of 5 × FAD mice during disease progression and triggering receptor expressed on myeloid cells-2 (TREM2) coexpression in microglia. **(A)**. Single-label immunohistochemistry (IHC) for the transcription factor PU.1 alone in the hippocampi of Alzheimer’s disease (AD) and control mice. Scale bar = 100 μm. **(B)** Representative western blot of PU.1 in whole hippocampal lysate, expressed as the mean ± S.E.M. **(C)** Analysis of the mRNA expression of PU.1 in the hippocampus from 1 to 18–20 months-old 5 × FAD mice and aged-matched wild type (WT) mice. The expression of PU.1 is presented as the mean ± S.E.M. and is indicated as the relative expression level via the 2^ΔΔCt^ method. The data were analyzed via one- or two-way analysis of variance followed by Bonferroni *post hoc* correction. #*p* < 0.05, ##*p* < 0.01, ###*p* < 0.001, 5 × FAD young group (3–4 months old) vs. the other 5 × FAD groups. *p < 0.05, **p < 0.01, ***p < 0.001, WT vs. 5 × FAD.

Our investigation of PU.1 expression within the hippocampus of 5 × FAD mice revealed significant increases in the protein and mRNA levels of PU.1. Immunohistological analysis revealed, parallel with aging, an increase in the number of PU.1-positive cells associated with reactive microgliosis in the CA1 region of the hippocampus (Figure 2A). Western blotting and quantitative PCR assays corroborated these findings, showing that both the protein and mRNA expression levels of PU.1 were markedly elevated in the 5 × FAD mice compared with those in the age-matched wild-type controls. The mRNA expression trend of PU.1 was similar to the protein expression trend, reflecting a highly consistent pattern of change (Figures 2B, C). Notably, the changes in the mRNA and protein expression tendencies of PU.1 were highly consistent with those of TREM2. In addition, the oldest 5 × FAD mice presented markedly higher PU.1 mRNA expression than did their young or middle-aged counterparts.

### 3.3 TREM2 expression is positively correlated with PU.1 expression in the brain microglia of 5 × FAD mice during chronic neurodegeneration

**FIGURE 3:**
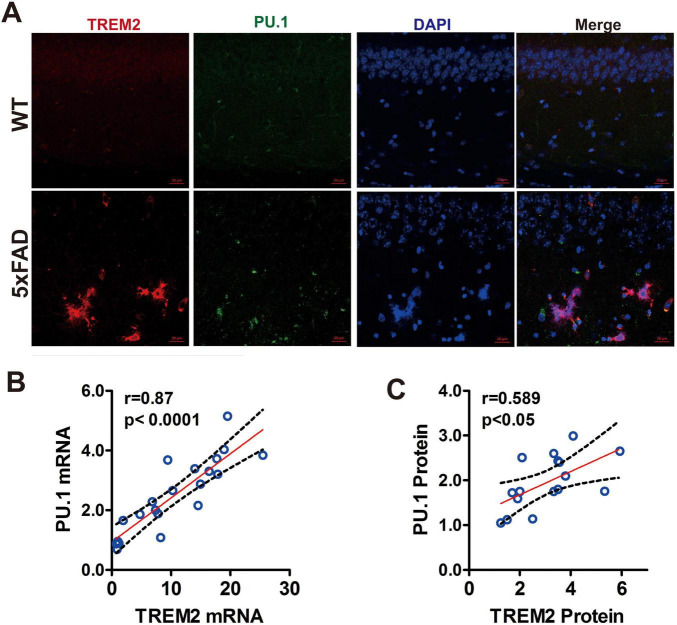
The positive correlation between triggering receptor expressed on myeloid cells-2 (TREM2) gene expression and PU.1 in the microglia of 5 × FAD mice during chronic neurodegeneration. **(A)** A significant correlation was found between the mRNA and protein expression of TREM2 and that of PU.1 in 5 × FAD mice across the age groups. **(B)** Linear regression analysis revealed that total TREM2 protein levels in the hippocampus were positively correlated with PU.1 levels. *p < 0.05, **p < 0.01, ***p < 0.001. **(C)** Analysis of coexpression of TREM2 (red) and PU.1 (green) in the hippocampi of 5 × FAD mice and aged-matched wild type (WT) mice by double immunofluorescence. Scale bar = 20 μm.

To elucidate the interplay between TREM2 and PU.1 within the microglial population in the brains of 5 × FAD mice, we used double-immunofluorescence labeling to visualize the coexpression of these markers. As depicted in Figure 3A, a one-to-one relationship was observed: nearly all of the TREM2-positive microglia also exhibited PU.1 positivity, and vice versa. This coexpression pattern was consistent in 5 × FAD and WT mice, suggesting a potential regulatory association between TREM2 and the transcription factor PU.1 in microglia. Pearson correlation analysis further substantiated this relationship in 5 × FAD mice, revealing a robust positive correlation between TREM2 and PU.1 expression (Figures 3B, C). These results suggest a possible mechanistic link whereby TREM2 elevation is closely tied to PU.1 upregulation during the course of chronic neurodegeneration.

### 3.4 β-Amyloid induces the upregulation of TREM2 and PU.1 expression in microglia *in vitro*

**FIGURE 4:**
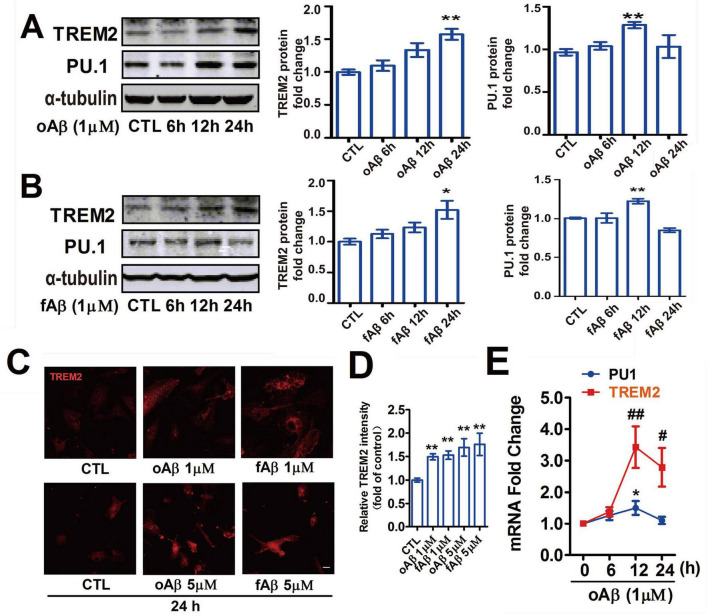
Aβ induced the upregulation of triggering receptor expressed on myeloid cells-2 (TREM2) and PU.1 expression in microglia *in vitro*. Treatment with 1 μM oAβ **(A)** or fAβ **(B)** was conducted for various durations in cultured BV-2 cells. **(C,D)** Representative single-label immunofluorescence staining of TREM2 in primary microglial cells after treatment with different doses of Aβ for 24 h. The values are expressed as the mean fluorescence intensity of TREM2. The data are expressed as relative fold changes compared with the controls. The values are expressed as the means ± S.E.M.s. Scale bar =10 μm. The data were analyzed via one-way analysis of variance followed by Bonferroni *post hoc* correction. **(E)** Quantitative real-time PCR analysis of TREM2 and PU.1 mRNA in primary microglia after 1 μM oAβ treatment at various time points. The expression of TREM2 is presented as the mean ± S.E.M. and is presented as the relative expression level according to the 2^ΔΔCt^ method. The data were analyzed via one-way analysis of variance followed by Bonferroni *post hoc* correction. Aβ, amyloid beta. *p < 0.05, **p < 0.01, ^#^*p* < 0.05, ^##^*p* < 0.01.

Given that 5 × FAD mice produce Aβ (1–42), which readily aggregates into oligomeric or fibrillar amyloid-beta throughout their life cycle, we utilized oligomeric Aβ (oAβ) and fibrillar Aβ (fAβ) as *in vitro* stimuli to mimic the *in vivo* scenario. On the basis of the analysis described above, we hypothesized that TREM2 expression upregulation in microglia might be influenced by the transcription factor PU.1 in an AD environment. We aimed to determine whether TREM2 and PU.1 were upregulated in cultured BV-2 or primary microglia after Aβ exposure. As demonstrated in Figures 4A, B, both oAβ and fAβ induced an increase in TREM2 expression in BV-2 microglia in a time-dependent manner. The various forms of Aβ induced a gradual increase in TREM2 expression, which was significant at 24 h after stimulation (Figures 4A, B). The expression of PU.1 increased mildly and peaked at 12 h after oAβ or fAβ treatment but then decreased at 24 h. Representative immunofluorescence images of TREM2 are depicted in cultured primary microglia in Figure 4C, with statistical analyses of three separate experiments presented in Figure 4D. This pattern was also confirmed in primary microglia by qRT–PCR (Figure 4E). These data demonstrate that both oAβ and fAβ contribute obviously to TREM2 upregulation in a time-dependent manner. Despite the slightly greater tendency observed in the oAβ group, no significant difference was evident between oAβ and fAβ.

### 3.5 PU.1 facilitates TREM2 mRNA and protein expression

**FIGURE 5:**
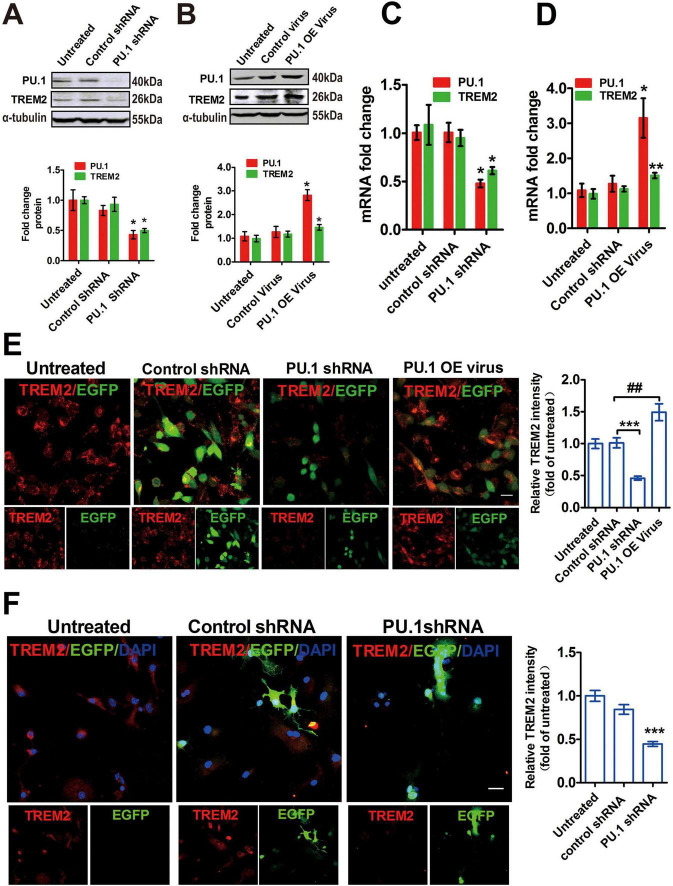
PU.1 promotes triggering receptor expressed on myeloid cells-2 (TREM2) gene expression at the mRNA and protein levels. **(A–D)** The effects of lentiviral particles on TREM2 and PU.1 gene expression in BV2 cells were confirmed by western blotting and qRT–PCR. The protein level is expressed as the relative level in the untreated cells, while the mRNA level is presented as the mean ± S.E.M. and is indicated as the relative expression level according to the 2^ΔΔCt^ method; *p < 0.05, **p < 0.01, ***p < 0.001, ^##^*p* < 0.01. The columns represent the results of the quantitative analysis of the expression of TREM2 and PU. **(E)** Immunofluorescence staining further revealed that PU.1 shRNA reduced the expression of TREM2 (red) and that the PU.1-overexpressing virus (PU.1 OE virus) increased the expression of TREM2. Scale bar = 10 μm. **(F)** The downregulation of TREM2 by PU.1 shRNA was also confirmed in primary microglia.Scale bar = 10 μm. EGFP = vector control. The values are expressed as the means ± S.E.M.s and were analyzed via ANOVA with Bonferroni *post hoc* correction. shRNA, short hairpin RNA.

The Chip-seq results revealed 5,264 target genes for the PU.1 protein, including TREM2 (Satoh et al., 2014), suggesting that TREM2 is a possible target gene of PU.1. We investigated the regulatory influence of PU.1 on TREM2 expression via shRNA in a lentiviral vector to suppress endogenous PU.1 expression in BV2 cells and primary microglia, as depicted in Supplementary Figure 1. Indeed, the suppression of PU.1 in BV2 cells resulted in a significant reduction in TREM2 expression at both the mRNA and protein levels (Figures 5A–C). This reduction was further verified by immunofluorescence staining (Figure 5E). Consistent with the BV2 cell data, the immunofluorescence data revealed a substantial PU.1 shRNA-induced decrease in the TREM2 protein level in primary microglia (Figure 5F). To ascertain whether the overexpression of PU.1 can increase TREM2 gene expression, we introduced lentiviral particles encoding PU.1 into BV2 cells, thus establishing an overexpression model that mimics microglia in 5 × FAD mice (Supplementary Figure 1). As expected, the overexpression of PU.1 significantly increased the expression of TREM2 at both the mRNA and protein levels (Figures 5B, D). The immunofluorescence results further supported our findings (Figures 5E, F). These findings indicate the crucial role of the PU.1 signal in modulating the expression of the TREM2 gene in mice.

### 3.6 PU.1 directly binds to the *TREM2* promoter or enhancer *in vivo* and regulates its promoter activity

**FIGURE 6:**
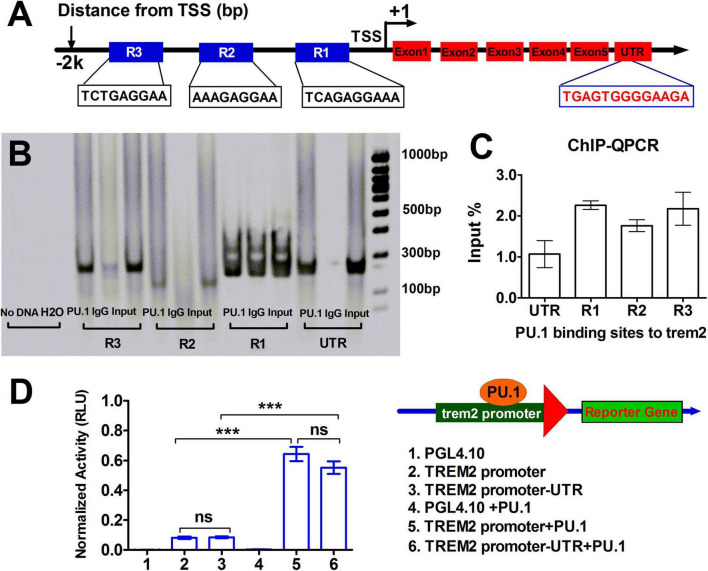
Direct binding between PU.1 and the triggering receptor expressed on myeloid cells-2 (TREM2) promoter or enhancer *in vitro* and *in vivo* and the regulation of the promoter activity of TREM2 The transcription factor PU.1 promoted TREM2 transcription. **(A)** Schematic representation of the TREM2 promoter sequence, which shows the sequences and positions of the four putative PU.1 binding sites highlighted in frame, used to study the binding site of the transcription factor PU.1 in the TREM2 promoter sequence. **(B,C)** A chromatin immunoprecipitation (ChIP) assay of microglial BV2 cells was performed using Abs against the PU.1 protein to immunoprecipitate chromatin from the cells. DNA fragments were analyzed by Quantitative real-time PCr (qRT–PCR) and are represented as percentages (%) input DNA **(C)**; a PAGE gel graph **(B)** of the qRT–PCR results after the ChIP assay for different binding sites from **(A)** is shown. **(D)** The 3.0 kb DNA sequence upstream of the transcription start site (TSS) was cloned and inserted into the pGL4.1 basic vector. The TREM2 promoter plasmid or TREM2 promoter containing UTR fragment constructs were transfected alone or cotransfected with the PU.1 expression plasmid into HEK293 cells via Lipofectamine 3000, and the pRL-TK Renilla vector expression vector pRL-SV40 was used for normalization. After 48 h of transfection, luciferase activity was measured with a dual-luciferase reporter assay system. The data are expressed as the relative luciferase units (RLUs) of three separate experiments. ***p < 0.001.

To ascertain whether PU.1 directly modulates TREM2 transcription, we utilized the ALGGEN Research Software^1^ online tool. Our analysis revealed that the basic structure of the *TREM2* promoter encompasses four PU.1 response elements. Three putative PU.1 binding site regions upstream of the transcription start site (TSS) are highlighted as R1, R2, and R3 in frame (Figure 6A). ChIP–qPCR on lysates extracted from BV2 cells supported these findings, revealing that PU.1 primarily binds to the initial putative site (P1) and the third binding site (P3) in the *TREM2* promoter and its 3′-UTR (Figures 6B, C).

To further investigate the impact of PU.1 on the promoter activity of *TREM2*, we conducted dual-luciferase reporter assays in HEK293T cells using relevant constructs. As shown in Figure 6D, when the *TREM2* promoter and *TREM2* promoter-UTR constructs were specifically transfected into HEK293 cells via Lipofectamine 3000, there was no marked variation in the luciferase activity. Nonetheless, an approximately 5-fold increase in luciferase activity was observed when these constructs were cotransfected with the PU.1-expressing plasmid. These compelling results suggest that PU.1 overexpression significantly increases the activity of the *TREM2* promoter via its cis-regulatory elements. Collectively, our findings provide strong evidence supporting the role of PU.1 as a transcriptional activator of the *TREM2* promoter, leading to elevated expression of TREM2 in activated microglia.

### 3.7 Aβ-mediated TREM2 upregulation in microglia depends on PU.1

**FIGURE 7:**
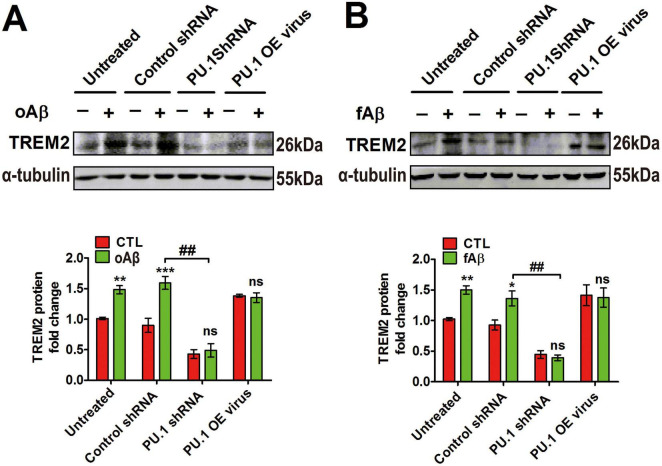
PU.1-dependent Aβ-mediated triggering receptor expressed on myeloid cells-2 (TREM2) expression upregulation in microglia. Microglial BV2 cells were transduced with a lentiviral vector encoding PU.1 short hairpin RNA (shRNA) or PU.1 sequences or control virus. After treatment with different viruses, the cells were treated with 1 μM oAβ **(A)** or fAβ **(B)** for 24 h. **(A,B)** Representative western blot of TREM2 protein levels upon oAβ or fAβ stimulation. Loss of PU.1 abrogated Aβ-induced TREM2 upregulation in microglia.oAβ, oligomeric amyloid beta; f Aβ, fiber amyloid beta. *p < 0.05, **p < 0.01, ***p < 0.001, ^##^*p* < 0.01.

To further elucidate the transcription factors contributing to the upregulation of TREM2 after Aβ stimulation, we directly targeted PU.1. The results revealed that the inhibition of PU.1 using shRNA considerably reduced the increase in TREM2 protein levels that was induced by both oAβ and fAβ (Figures 7A, B). Intriguingly, PU.1 overexpression failed to amplify the effects of oAβ and fAβ, implying that this transcription factor may exhibit a threshold effect or depend on additional cofactors to increase its activity. These observations suggest that PU.1 plays a critical role in the oAβ- and fAβ-mediated upregulation of TREM2 in BV2 microglia and may serve as a transcriptional activator tethered to the *TREM2* promoter.

### 3.8 The knockdown of both TREM2 and PU.1 impairs microglial phagocytosis *in vitro*

**FIGURE 8:**
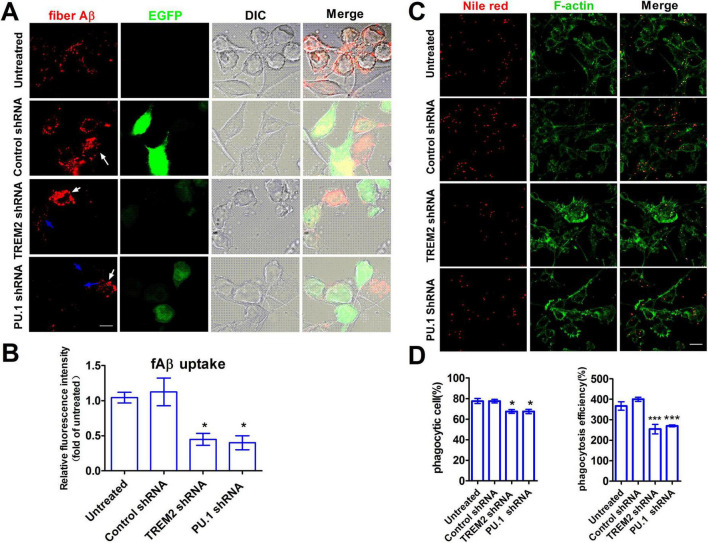
The impairment of microglial phagocytosis by triggering receptor expressed on myeloid cells-2 (TREM2) and PU.1 knockdown *in vitro*. **(A)** For the Aβ peptide phagocytosis assay, the cells were incubated alone with Hilyte-555-labeled fAβ (1–42) for 1 h (1.0 μM). After viral transduction, both TREM2 and PU.1 knockdown reduced microglial phagocytosis of fAβ (1–42). The mean fluorescence intensity **(B)** was quantified and is presented as the mean ± S.E.M. **(C)** For the microsphere phagocytosis assay, the cells were treated with Nile red microspheres (red) for 30 min. The cells were stained with phalloidin to visualize F-actin (green), and the percentages of microglia that underwent phagocytosis **(C)** and phagocytic efficiency **(D)** are presented as the means ± S.E.M.s (**p < 0.01). Scale bar = 10 μm. The data were quantified relative to those of the untreated group and analyzed via ANOVA with Bonferroni *post hoc* correction. *p < 0.05, ***p < 0.001, knockdown group versus control virus group.

One of the significant pathological characteristics of AD is the heightened accumulation of Aβ. The upregulation of TREM2 and PU.1 expression may play a substantial role in this context by enhancing the phagocytosis of Aβ and hampering amyloid aggregation. We suppressed TREM2 and PU.1 in BV2 cells using RNAi in lentiviral particles and performed phagocytosis assays to further substantiate their neuroprotective effects. As depicted in Figure 8, the microglial phagocytic response was noticeably inhibited. Specifically, in the Aβ42 phagocytosis assay, BV2 cells were incubated with Hilyte-555-labeled fAβ (1–42) for 1 h (1.0 μM) after stable viral transduction, and the mean fluorescence intensity associated with the cells was determined (Figures 8A, B). The results revealed that, in both knockdown groups, the cells transduced with the virus (indicated by the blue arrow, EGFP immune positive) contained minimal fluorescent fAβ, whereas a significant quantity of fAβ was internalized by the untransduced cells (indicated by the white arrow, EGFP immune negative) (Figure 8A). Compared with the control virus group, the TREM2 shRNA and PU.1 shRNA groups presented decreases in the uptake of labeled fAβ of approximately 55% and 50%, respectively (Figure 8B). Compared with untreated or control shRNA-treated BV2 cells, microglial BV2 cells treated with TREM2 shRNA or PU.1 shRNA demonstrated significantly decreased phagocytosis of Nile red fluorescent microspheres, reducing the phagocytic efficiency by approximately 33% and 30%, respectively. Notably, the percentage of phagocytic cells remained constant in the TREM2- and PU.1-knockdown groups (Figures 8C, D). These findings suggest that the Aβ-induced expression of TREM2 and PU.1 in microglia may enhance the clearance of Aβ42 peptides, serving as a compensatory mechanism in the Aβ-enriched environment characteristic of AD.

Moreover, we overexpressed PU.1 in BV2 cells. Compared to control cells, BV2 cells with PU.1 overexpression exhibited an increasing trend in Aβ phagocytosis efficiency, Nile red fluorescent microsphere phagocytosis efficiency, and the percentage of phagocytic cells. However, the differences in Aβ phagocytosis efficiency and the percentage of phagocytic cells for Nile red fluorescent microspheres were not statistically significant (Supplementary Figures 2, 3).

## 4 Discussion

In the present study, we observed an age-related increase in TREM2 and PU.1 expression at both the mRNA and protein levels in 5 × FAD mice. Notably, for the first time, we demonstrated a positive correlation between TREM2 and PU.1 gene expression in microglia across all stages of disease progression.

Our observations revealed that the incremental increase in TREM2 and PU.1 expression corresponds with increasing Aβ accumulation in the brains of younger and older mice. Consistent with the findings of previous studies, we observed no change in TREM2 and PU.1 expression between 5 × FAD and WT mice at 1 month of age, a stage that precedes significant intraneuronal Aβ42 accumulation (Jay et al., 2015; Oakley et al., 2006).

Microglia, inherent immune cells in the brain, maintain tissue homeostasis and the innate immune balance by detecting pathological changes within the CNS (Brioschi et al., 2023; Hickman and El Khoury, 2014; Shi et al., 2023; Zhang et al., 2023). Our results suggest a time-related increase in TREM2 expression during AD progression, potentially compensating for inadequate microglial immune responses. As an important marker of the M2 phenotype, the upregulation of TREM2 expression exerts numerous wound-healing functions, such as antioxidative stress activities, the clearance of apoptotic cells or debris, anti-inflammatory activities, and amyloid decumulation (Filipello et al., 2022; Li H. et al., 2022; Li R. Y. et al., 2022; Liebold et al., 2023; Liu et al., 2020; Zhao et al., 2022a; Zhao et al., 2022b).

Our study revealed that different forms of Aβ42, such as oAβ and fAβ, considerably upregulated TREM2 expression in a PU.1-dependent manner. Although numerous studies have reported that TREM2 expression is upregulated in amyloid plaque-associated microglia *in vivo* (Frank et al., 2008; Jay et al., 2015), our research is the first to show that PU.1 knockdown abrogates this phenomenon *in vitro*. We identified PU.1 as a key regulator of TREM2 expression. Further, PU.1 overexpression amplified endogenous TREM2 mRNA and protein levels, which were reduced by PU.1 knockdown, establishing PU.1 as a transcriptional determinant of Aβ-induced TREM2 expression upregulation at the cellular and molecular levels.

We discovered that TREM2 and PU.1 knockdown impaired microglial phagocytosis *in vitro*, reinforcing the notion that PU.1 modulates TREM2 expression and, in turn, regulates microglial phagocytosis. In the context of TREM2 knockdown, impaired phagocytic function aligns with functional activity loss, which is supported by earlier studies exploring the consequences of *TREM2* deletion and *TREM2* missense mutations such as R47H, Y38C, and T66M (Sayed et al., 2021). The persistent upregulation of TREM2 and PU.1 expression in 5 × FAD mice may represent a defense response of the innate immune system to counterbalance amyloid plaque accumulation in the AD brain (Lue et al., 2015). However, as AD progresses, clearance mechanisms may fail to adapt to amyloid overproduction (Rivest, 2015).

Using molecular biological techniques such as ChIP, EMSA (data not shown), and luciferase reporter assays, we demonstrated that PU.1 directly binds to a probable promoter or enhancer region of *TREM2*. Our study further established that coexpressing exogenous PU.1 promoted the activity of the *TREM2* promoter, reinforcing the hypothesis that PU.1 transcriptionally controls *TREM2* gene expression, driving the transition in the functional state of microglia, resulting in increased Aβ clearance (Lu et al., 2023). These findings indicate that the murine *TREM2* gene is a downstream component of PU.1 signaling.

Our study elucidates a novel PU.1-mediated transcriptional regulation mechanism governing TREM2 expression, demonstrating its pivotal role in modulating microglial activation dynamics during the progression of Alzheimer’s disease. A recent study suggested that the regulation of TREM2 may involve complex transcriptional controls, with RXR activation enhancing bexarotene-mediated Trem2 transcription in APP/PS1 mice (Rivest, 2015). Other transcription factors may participate in Aβ-mediated TREM2 expression (He et al., 2022; Lu et al., 2023). Indeed, PU.1 is a crucial regulator of the development and proliferation of microglia (Kierdorf et al., 2013; Speicher et al., 2022). Both in previous studies and in this study, microglia in the brains of 5 × FAD mice were found to be continuously activated and to proliferate with increasing cognitive dysfunction during aging (He et al., 2023; Wei et al., 2016). While this effect may promote Aβ phagocytosis and clearance (Lau et al., 2020), it is also potentially associated with an increased inflammatory response that may cause neuronal damage and thus accelerate cognitive decline (Wei et al., 2016). Therefore, targeted modulation of PU.1 is an important direction for future AD intervention (Cakir et al., 2022; Rustenhoven et al., 2018; Wei et al., 2016).

While our study provides valuable insights, some limitations remain. First, our findings pinpoint PU.1 as a major regulator of *TREM2* gene expression without considering other transcription factors that may play consequential roles in Aβ-mediated TREM2 expression. This highlights the need for further experiments to investigate potential coregulators. Second, the current siRNA-mediated knockdown approach, while informative, does not fully recapitulate the complete genetic deletion of PU.1. A floxed PU.1 mouse model would offer a more definitive genetic approach to validate our findings, providing more robust *in vivo* confirmation of the transcriptional regulation of TREM2 by PU.1. In future studies, conditional knockout strategies should be used to comprehensively explore the genetic mechanisms underlying the function of PU.1 in microglial biology and AD progression. Third, the BV2 microglial cell line are widely used alternatives to primary cells because they are more readily available in neuroscience research of microglia biology. Studies using BV2 cells as *in vitro* models of microglia have provided insights into the signaling pathways such as microglial activation and polarization, influencing the inflammatory response and phagocytic activity regulated by TREM2 (Chen et al., 2023; Shi et al., 2024). However, previous studies have reported detectable TREM2 expression in BV2 cells but not in HMC3 (an *in vitro* microglial model) or B6 (SHIP1 knockout) primary microglial cells (Ahat et al., 2024). In contrast, our experiments demonstrated high TREM2 expression in both primary microglial cells and BV2 cells. While proteomic analyses indicate substantial similarity between mouse primary microglia and BV2 cells, with only minimal differences in protein expression levels, it is critical to acknowledge that BV2 cells fail to fully recapitulate the gene expression patterns or epigenetic features of brain-derived primary microglia. Primary microglia exhibit a more complex and dynamic transcriptional profile that closely mirrors their *in vivo* state within the CNS. In comparison, immortalized BV2 cells—despite expressing PU.1—lack key characteristics of primary microglia, such as the capacity to replicate the heterogeneity and functional diversity observed *in vivo* (He et al., 2018; Pimenova et al., 2021).

A limitation of our study lies in its heavy reliance on BV2 cells for mechanistic investigations of PU.1-mediated phenotypic characterization. While this approach provides valuable preliminary insights, future *in vivo* studies will be essential to validate these findings in the context of AD pathophysiology. Additionally, strategies incorporating small-molecule phenotypic screening for AD drug discovery should prioritize models that better reflect the biological complexity of human microglia.

## 5 Conclusion

**FIGURE 9:**
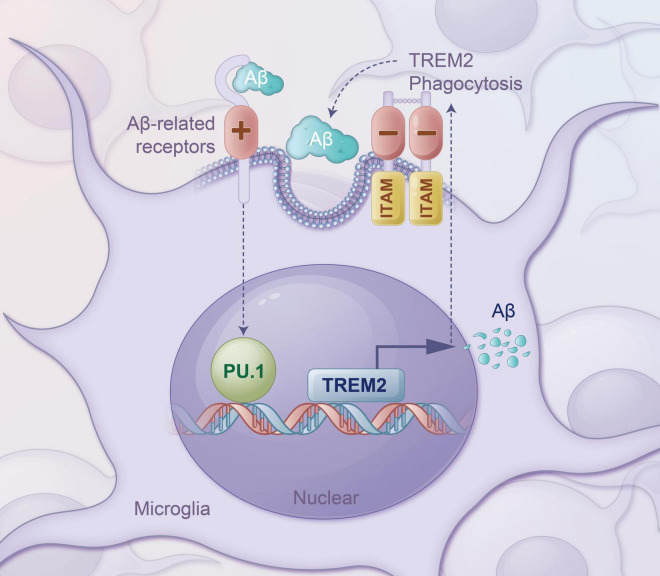
Main schematic overview and working models used in this study.

In summary, we provide strong evidence for the transcriptional regulation of TREM2 expression by PU.1 in activated microglia in the Aβ milieu (Figure 9). These results indicate that PU.1/Spi1 plays a key role in regulating specialized gene expression and mediates microglial phenotype or functional shifting during AD pathogenesis and progression. Modulating aberrant PU.1 signaling pathways may offer innovative strategies to manipulate neuroimmune interactions and ultimately provide promising therapeutic targets for AD treatment.

## Data Availability

The original contributions presented in this study are included in this article/Supplementary material, further inquiries can be directed to the corresponding authors.

## Footnotes

1
http://alggen.lsi.upc.edu/
