# Supplementary material for: PU.1 dictates β-amyloid-induced TREM2 expression upregulation in microglia in a transgenic model of Alzheimer’s disease
Source: Front Aging Neurosci. 2025 May 1;17:1537388. doi: 10.3389/fnagi.2025.1537388 (PMC12078285; doi:10.3389/fnagi.2025.1537388)
Supplement: Supplementary file 1 [file Data_Sheet_1.pdf]

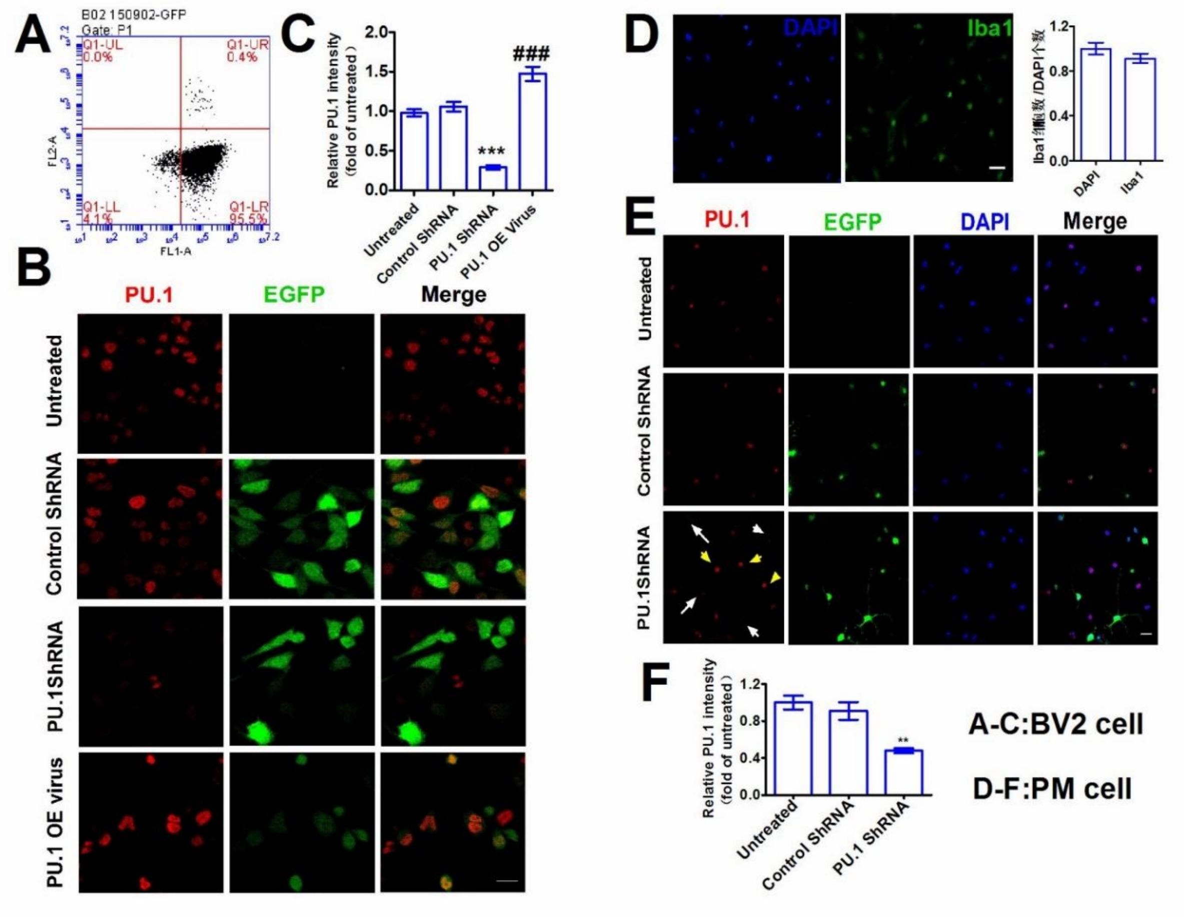

Supplementary Figure 1. The establishment of cell models of KD or OE of transcription factor PU.1 by PU.1 silencing RNA and PU.1 overexpression .

(A) EGFP of BV2 cell was performed to detect transfection efficiency by flow cytometry after transducing with control lentiviral vectors. (B-C), PU.1 protein levels of BV2 cells with different treatments were detected by cellular immunofluorescence staining analysis. Columns represent quantitative analysis of mean fluorescence intensity of PU.1. n=3, **p*<0.05, ****p*<0.001, The scale bar = 10 μm. (D) The purity of primary microglia was identified by Iba1 (green) immunofluorescence staining. Cell nuclei were counterstained with DAPI (blue). Scale bar = 20 μm. Columns showed the ratio of the number of Iba1-positive cells to that of DAPI-positive cells. (E-F) PU.1 protein levels of primary microglial cells with different treatments were detected by cellular immunofluorescence staining. Columns represent quantitative analysis of mean fluorescence intensity of PU.1. n = 3, ***p*<0.01. Scale bar = 20 μm

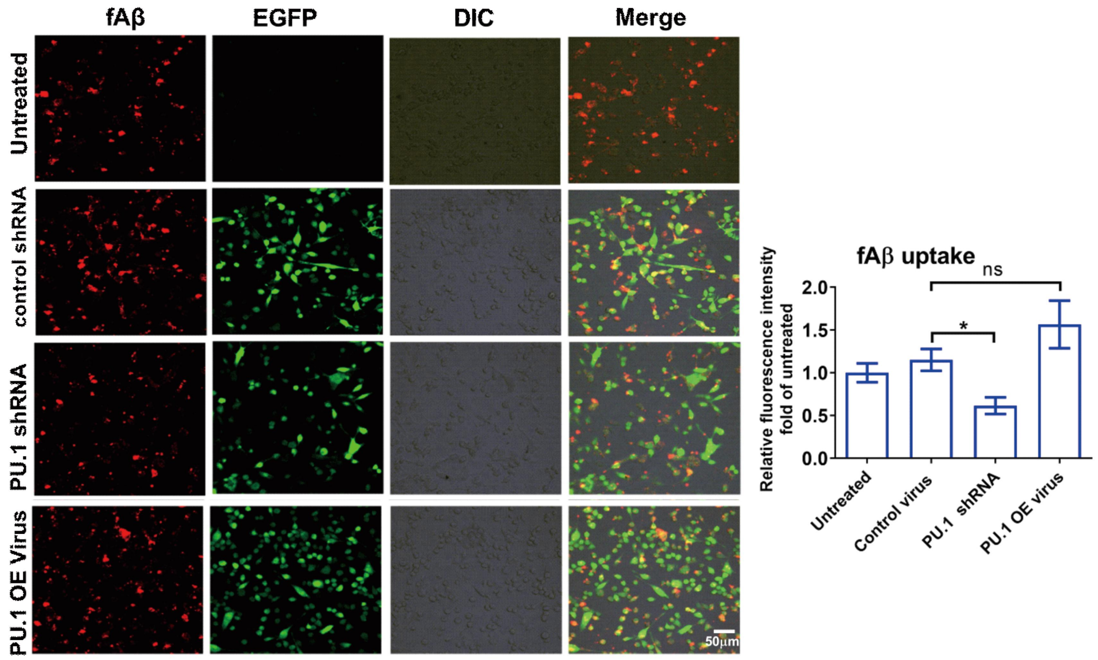

Supplementary Figure 2. Aβ phagocytosis assay following PU.1 overexpression. To evaluate the effect of PU.1 overexpression on the phagocytosis of Aβ in microglia, we conducted the Aβ peptide phagocytosis assay after inducing the overexpression of PU.1. After viral transduction, the BV2 cells were incubated alone with Hilyte-555-labeled fAβ (1-42) for 1 hour (1.0 μM). BV2 cells with PU.1 overexpression exhibited an increasing trend in Aβ phagocytosis, while PU.1 knock-down inhibited the phagocytic function of fAβ. The mean fluorescence intensity was quantified and is presented as the mean ± S.E.M. n = 3, Scale bar = 50 μm.

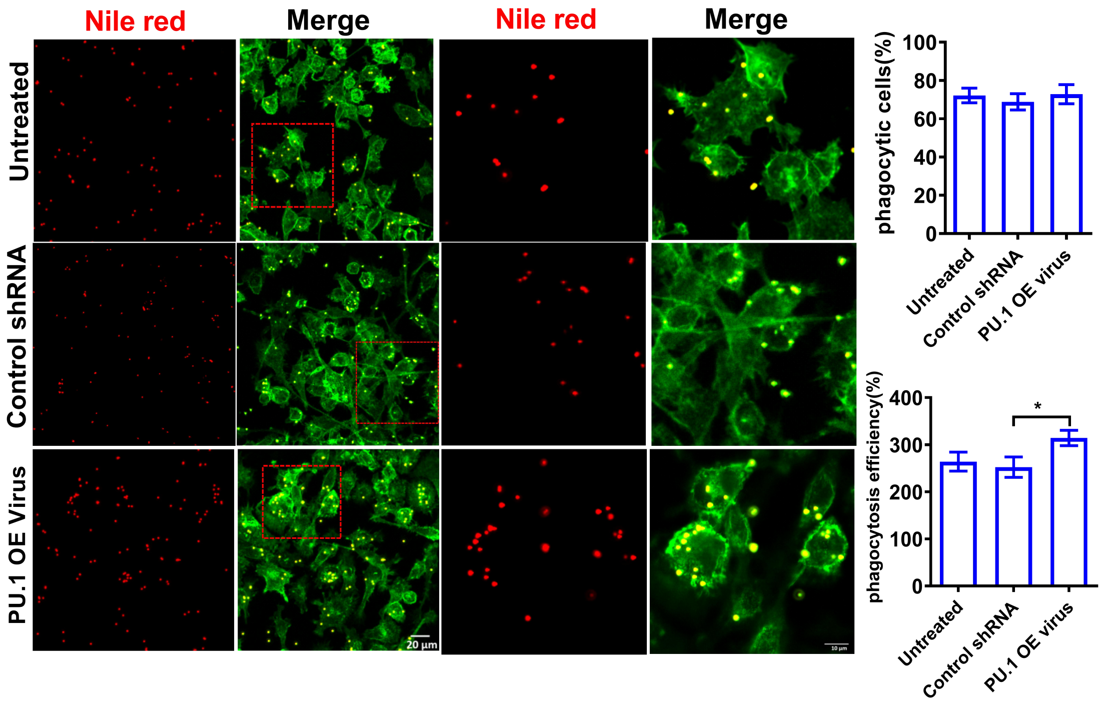

Supplementary Figure 3. Enhanced phagocytosis of Nile Red Fluorescent microspheres following PU.1 overexpression

For evaluate the effect of PU.1 overexpression on the phagocytosis of microglia, we conducted the the microsphere phagocytosis assay after inducing the overexpression of PU.1, the cells were treated with Nile red microspheres (red) for 30 min. The cells were stained with phalloidin to visualize F-actin (green), and the percentages of microglia that underwent phagocytosis and phagocytic efficiency are presented as the means ± S.E.M. (*$p<0.05$). The two columns on the right respectively correspond to the enlarged content within the dashed-line boxes of the pictures on their left sides. n = 3, Scale bar = 20 μm（the left two columns）or Scale bar = 10 μm（the right two columns）.
